# Development of an Image-Guided Orthotopic Xenograft Mouse Model of Endometrial Cancer with Controllable Estrogen Exposure

**DOI:** 10.3390/ijms19092547

**Published:** 2018-08-28

**Authors:** Gonda FJ Konings, Niina Saarinen, Bert Delvoux, Loes Kooreman, Pasi Koskimies, Camilla Krakstad, Kristine E. Fasmer, Ingfrid S. Haldorsen, Amina Zaffagnini, Merja R. Häkkinen, Seppo Auriola, Ludwig Dubois, Natasja Lieuwes, Frank Verhaegen, Lotte EJR Schyns, Roy FPM Kruitwagen, ENITEC Consortium, Sofia Xanthoulea, Andrea Romano

**Affiliations:** 1 GROW—School for Oncology & Developmental Biology, Maastricht University, 6229HX Maastricht, The Netherlands; bert.delvoux@maastrichtuniversity.nl (B.D.); loes.kooreman@mumc.nl (L.K.); amina.zaffagnini@studio.unibo.it (A.Z.); ludwig.dubois@maastrichtuniversity.nl (L.D.); n.lieuwes@maastrichtuniversity.nl (N.L.); frank.verhaegen@maastro.nl (F.V.); Lotte.schyns@maastro.nl (L.E.J.R.S.); r.kruitwagen@mumc.nl (R.F.P.M.K.); sofia.xanthoulea@maastrichtuniversity.nl (S.X.); a.romano@maastrichtuniversity.nl (A.R.); 2 Department of Obstetrics and Gynaecology, Maastricht University Medical Centre, P. Debyelaan 25, 6229HX Maastricht, The Netherlands; 3 Forendo Pharma Ltd., FI-20520 Turku, Finland; niina.saarinen-aaltonen@forendo.com (N.S.); Pasi.Koskimies@forendo.com (P.K.); 4 Institute of Biomedicine, Research Centre for Integrative Physiology and Pharmacology and Turku Center for Disease Modeling (TCDM), University of Turku, FI-20520 Turku, Finland; 5 Department of Pathology, Maastricht University Medical Centre, 6229HX Maastricht, The Netherlands; 6 Department of Obstetrics and Gynaecology, Haukeland University Hospital, 5021 Bergen, Norway; camilla.krakstad@med.uib.no; 7 Centre for Cancer Biomarkers, Department of Clinical Science, University of Bergen, 5021 Bergen, Norway; 8 Department of Radiology, Haukeland University Hospital, 5021 Bergen, Norway; kristine.eldevik.fasmer@helse-bergen.no (K.E.F.); ingfrid.helene.salvesen.haldorsen@helse-bergen.no (I.S.H.); 9 Section for Radiology, Department of Clinical Medicine, University of Bergen, 5020 Bergen, Norway; 10 School of Pharmacy, University of Eastern Finland, FI-80101 Kuopio, Finland; merja.hakkinen@uef.fi (M.R.H.); seppo.auriola@uef.fi (S.A.); 11 Department of Radiotherapy (MAASTRO), Maastricht University, 6229HX Maastricht, The Netherlands

**Keywords:** endometrial cancer, orthotopic xenograft model, estrogen dependent, bioluminescence imaging, contrast-enhanced CT scan

## Abstract

Endometrial cancer (EC) is the most common gynaecological malignancy in Western society and the majority of cases are estrogen dependent. While endocrine drugs proved to be of insufficient therapeutic value in the past, recent clinical research shows promising results by using combinational regimens and pre-clinical studies and identified potential novel endocrine targets. Relevant pre-clinical models can accelerate research in this area. In the present study we describe an orthotopic and estrogen dependent xenograft mouse model of EC. Tumours were induced in one uterine horn of female athymic nude mice using the well-differentiated human endometrial adenocarcinoma Ishikawa cell line—modified to express the luciferase gene for bioluminescence imaging (BLI). BLI and contrast-enhanced computed-tomograph (CE-CT) were used to measure non-invasive tumour growth. Controlled estrogen exposure was achieved by the use of MedRod implants releasing 1.5 μg/d of 17β-estradiol (E2) in ovariectomized mice. Stable E2 serum concentration was demonstrated by LC-MS/MS. Induced tumours were E2 responsive as increased tumour growth was observed in the presence of E2 but not placebo, assessed by BLI, CE-CT, and tumour weight at sacrifice. Metastatic spread was assessed macroscopically by BLI and histology and was seen in the peritoneal cavity, in the lymphovascular space, and in the thoracic cavity. In conclusion, we developed an orthotopic xenograft mouse model of EC that exhibits the most relevant features of human disease, regarding metastatic spread and estrogen dependency. This model offers an easy to manipulate estrogen dosage (by simply adjusting the MedRod implant length), image-guided monitoring of tumour growth, and objectively measurable endpoints (including tumour weight). This is an excellent in vivo tool to further explore endocrine drug regimens and novel endocrine drug targets for EC.

## 1. Introduction

Endometrial cancer (EC) is the most common gynaecological malignancy in the Western world with over 60,000 new cases estimated in 2017 (U.S.; https://seer.cancer.gov). About 70% of ECs are diagnosed at an early stage and are treated with surgery and with selected cases also receiving adjuvant chemo/radio therapy. Nevertheless, about 25% of EC patients will develop recurrent diseases. In patients with recurrent EC, and in EC cases with primary advanced stage and distant metastatic disease, treatment options are limited. They consist of aggressive chemotherapy, which however yields low efficacy, poor patient prognosis, and is accompanied by significant side effects [1,2]. Hence, there is a need for additional treatment possibilities.

EC is an estrogen dependent disease, but in contrast to other hormone-dependent conditions like prostate or breast cancer where endocrine therapy is successfully used, treatment of EC with hormonal drugs showed little efficacy [3,4,5,6]. However, recent clinical trials indicate that the efficacy of hormonal drugs is improved if combined with other targeted treatments (see Section 3) [7]. Since ongoing pre-clinical research has already identified a number of additional novel endocrine targets [8,9,10], it can be envisaged that in the near future several endocrine targets and novel drugs will be available for various combinational regimens in EC clinical trials.

To accelerate research in this field, relevant pre-clinical models need to be developed that better mimic the human situation, in comparison with the current models. In the case of EC, pre-clinical models are based on in vitro 2D monolayer cell cultures, 3D spheroid cell cultures, and in vivo models using the chorioallantoic membrane assay (CAM) or by a subcutaneous injection of human EC cell lines in athymic mice. However, these models are suboptimal and often lack estrogen dependency and clear estrogen responses (see Section 3) [10,11,12].

Orthotopic xenografts models are relevant pre-clinical tools because the tumour is induced at the same location and similar microenvironment where it occurs in humans. Recently, different research groups reported successful orthotopic EC xenografts in mice that mimicked the human condition including metastatic spread [13,14,15,16]. In particular, Haldorsen and co-workers optimised the orthotopic EC xenograft using the estrogen dependent Ishikawa cell line. However, tumours were grown in the presence of the natural endogenous source of estrogens, the ovaries, and the authors did not explore the possibility to control estrogen exposure [14]. To be able to control and modulate steroidal exposure and mimic conditions like post menopause, in collaboration with the same authors, we here further advanced this orthotopic EC xenograft model to control steroid exposure and induce estrogen dependent tumour growth—as is the case in the majority of ECs in humans. This model offers the possibility to manipulate estrogen (and other steroid) dosage, image-guided monitoring of tumour growth, and easily determined objective endpoints to measure estrogen response. Moreover, it exhibits clinical features similar to human EC like metastatic spread and the presence of lymphovascular invasion (LVI), thus being a highly relevant model of the disease. We believe this is an excellent in vivo tool to further explore endocrine drug regimen and novel endocrine drug targets for EC.

## 2. Results

### 2.1. Orthotopic EC Xenograft: Cell Titre Optimization

**Figure 1:**
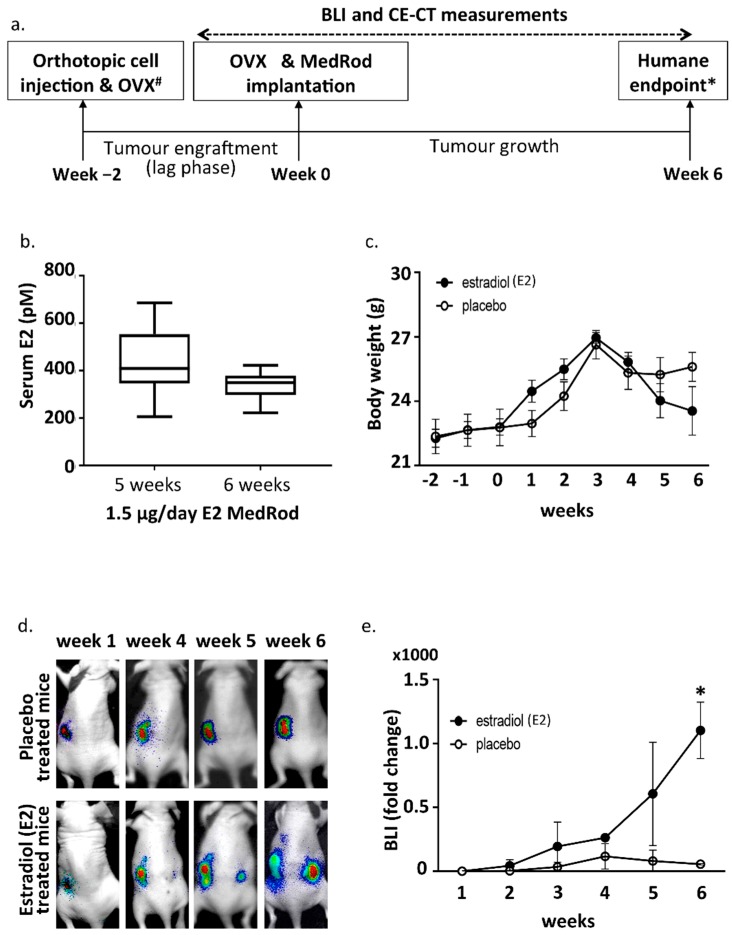
Experimental design and bioluminescence (BLI) results. (**a**) Timeline of the mouse model. Tumors were induced by orthotopic cell injection in one horn of the uterus (week 2). At the same time, ipsilateral OVX was performed. Two weeks later (i.e., at the end of the lag phase; week 0) OVX was performed at the other uterine horn as well and the MedRod delivery system for E2 or placebo supplementation was implanted subcutaneously. Tumor growth was monitored weekly by BLI until the humane endpoint was reached. Before euthanasia CE-CT was performed. ^#^ Ipsilateral OVX and OVX was not performed in the first experiment testing the cell titer. * Humane endpoint: signs of discomfort due to large-sized tumours. (**b**) Serum E2 concentration (LC-MS/MS) in mice implanted with MedRod devices releasing 1.5 μg/day of E2. Boxplots indicate the median and the lower and upper quartiles. Blots represent data from nine (5 weeks) and 12 mice (6 weeks) per time point. (**c**) Body weight. Mean values and standard deviations are shown (placebo: 8 mice; E2: 8 mice). (**d**) Representative images of in vivo sequential BLI. Note that the BLI signal from the location of primary tumour induction tends to decrease at late time points due to necrosis of the tumour tissue (as assessed histologically). (**e**) BLI fold change during the experiment. Shown data refers to the experiments with the Ishikawa clone three. Similar data was obtained with the other cell clones (data not shown). Mean values and standard deviations are shown (placebo: 3 mice; E2: 2 mice). Asterisk (*) indicates a *p*-value < 0.05 (*t*-test).

We first determined the optimal number of Ishikawa-derived cells required for tumour induction using three different titres (1 × 10^6^, 3 × 10^6^ and 5 × 10^6^ cells/mouse; Table 1) that were injected in gonad-intact mice with no further steroid supplementation (see scheme in Figure 1a). Ishikawa clone 1 was used in this optimization. The lowest dose (1 × 10^6^ cells) gave a weak bioluminescence (BLI) signal and tumour engraftment did not occur in one-out-of-three injected mice. Although all mice receiving 5 × 10^6^ cells developed a tumour, it was technically challenging to inject such a large number of cells into the uterus. Orthotopic injection of 3 × 10^6^ cells appeared to be the optimal dose since no technical difficulties were experienced and all mice developed a tumour.

### 2.2. Ovariectomy and Estrogen Supplementation (MedRod Implants)

In order to control the levels of circulating E2 and measure estrogen dependent EC growth, the ovaries, which are the main endogenous sources of female sex steroids, were removed by ovariectomy (OVX) and E2 was provided exogenously by E2 releasing MedRod implants. MedRods consisted of 1–4 mm diameter cylinder-shaped elastic rods containing E2 and were implanted subcutaneously on the back of the mice, in between the scapulae. The serum E2 concentrations in mice with E2 MedRods with daily releases of 1.5 μg/day (5.5 nmol/day) resulted in constant average serum concentrations of E2 being between 338 and 443 pM (Figure 1b). Accordingly, implants releasing another kind of estrogen (estrone) had similar performances, i.e., constant serum levels of the test compound at different doses (Supplementary Figure S1).

### 2.3. Estrogen Responsiveness of the Orthotopic EC Xenograft

To assess estrogen dependency of tumour growth, cancer cells (3 × 10^6^) were injected, mice were ovariectomized, and E2 or placebo releasing MedRods were implanted (Figure 1a and Table 2). Three Ishikawa derived clones (clone 1, clone 2 and clone 3) were used. These clones were obtained through genetic modification and clonal expansion of the parental Ishikawa cells (see Section 4) and behave similarly in terms of E2 responsiveness based on previous characterisation [10]. Three clones were nevertheless used to avoid any clonal effect biasing the results.

Previous studies on estrogen dependent cancer models showed that estrogens are required for initial tumour engraftment (lag phase) [17,18,19]. Therefore, complete OVX was performed approximately two weeks after tumour cell injection, i.e., when tumours were successfully engrafted, as assessed by positive BLI signals. However, at the time of tumour cell injection, the ovary at the same horn-side of the injection was removed (ipsilateral OVX) to avoid that, after the lag phase (two weeks later), tumour overgrowth would impede the complete removal of the ovary or that part of the tumour would be removed together with the ovary. Following complete OVX, MedRod implants releasing either placebo or 1.5 μg E2/day were implanted (see timeline in Figure 1a and the procedure in Supplemental Figure S2).

Tumors were grown for an additional six weeks (eight weeks in total; i.e., two weeks for tumour engraftment/lag phase and six weeks for E2-stimulated tumour growth) and growth was monitored weekly by BLI. Tumors developed in all sixteen mice (Table 2). Three weeks after OVX and E2/placebo supplementation, weight loss started to be observed in all mice, most likely because of the side effects and initial cachexia due to tumour growth, and this was more pronounced in the E2 group (Figure 1c). At week six after OVX and E2/placebo supplementation, mice presented signs of discomfort due to large-sized tumours and mice were euthanized (humane endpoint). During the six weeks following OVX and E2/placebo supplementation, tumour growth (assessed by BLI) was clearly E2 responsive and in the E2 arm, the BLI signal was not limited to the tumour induction area (as in the placebo group), but it spread to other locations in the abdomen (Figure 1d,e). As expected, within the same treatment group (E2 or placebo), no differences were observed in the growth of tumours induced with different Ishikawa clones.

### 2.4. CE-CT, Endpoint and Tumor Characteristics

**Figure 2:**
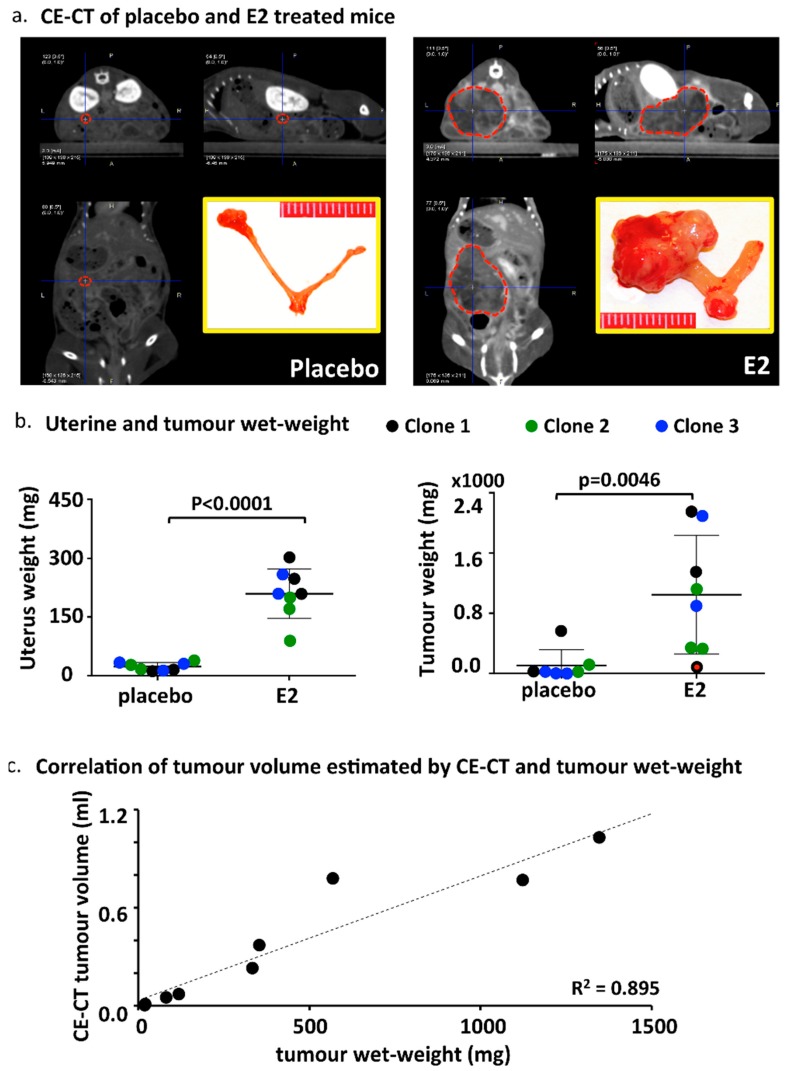
Tumor growth determined by CE-CT and wet-weight at sacrifice. (**a**) Representative CE-CT scan images centered by the blue cross at the center of the tumour (indicated by the red dotted line). The yellow-bordered inset shows the size of the uterus and the tumour surgically removed at sacrifice. (**b**) Uterine and tumour wet-weight in the E2 treated and placebo groups. The color codes indicate the different cell clones used. The red dot in the right panel shows the tumour weight of an E2 treated mouse from clone 1 with no proper tumour engraftment (no BLI signal at the moment of E2/placebo supplementation, week 0) probably due to sub-optimal cell injection (see also Table 3). *p* values are computed using *t*-test (outlier included). (**c**) Correlation between tumour volume estimated by CE-CT and the wet-weight of surgically removed tumours at sacrifice.

**Figure 3:**
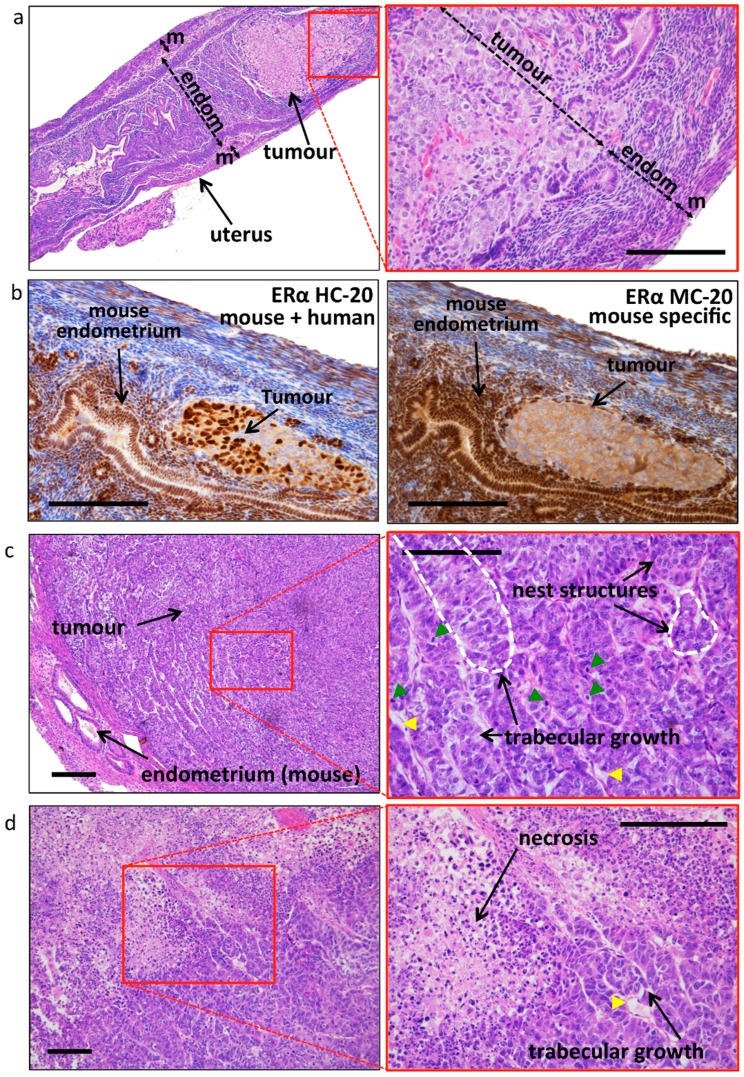
Tumor histology and estrogen receptor expression. (**a**) Representative histology showing the orthotopic location of the induced tumour. The mouse shown is from a placebo treated mouse, where tumour growth remained confined inside the uterus until the end of the experiment. The thickness of the endometrium (endom.) and the myometrium (m) (and the tumour, in the right image only) is indicated by the double-headed arrow. Bar scale: 200 μm. (**b**) Analysis of ER-α expression using antisera directed against human receptor but cross-reacting with the mouse ER-α (left, HC-20), where both tumour (most likely of human origin) and mouse endometrial tissues have nuclear staining, and using antisera directed against mouse ER-α (right, MC-20), with nuclear staining only in mouse tissues and showing cytoplasmic background in the tumour. Bar scale: 200 μm. (**c**) Representative image of the tumour histology (E2 treated sample), with nest and trabecular structures (indicated by the dotted white line on the enlargement, right image). Mitotic figures (green arrowheads) and blood vessels (yellow arrowheads) are visible. Bar scale: 200 μm. (**d**) Representative image of a large tumour with a large necrotic core. Yellow arrowheads: blood vessels. Bar scale: 200 μm.

Prior to sacrifice (six weeks after OVX and E2/placebo supplementation), 11 animals were investigated by CE-CT scans (Table 3 and Figure 2a). Tumor volume estimation confirmed a more sustained tumour growth in the E2 group (mean volume 0.5 mL) compared with the placebo group (mean volume 0.2 mL). Tumor density was very variable between mice (Table 3) and was not significantly different between the groups (E2 group: 28 ± 26 vs. placebo group: 92 ± 67). Mice were euthanized and uteri, tumours, and other organs were isolated and examined. As expected, the uteri of the E2 group were substantially larger compared with the placebo, due to estrogen stimulation (Supplemental Figure S2). Both uterine wet-weight as well as tumour wet-weight were significantly higher in the E2 treated group compared with placebo (Figure 2b). The weight of surgically removed tumours correlated with the tumour volume estimated by CE-CT (Figure 2c). Histologic evaluation of uteri confirmed the presence of epithelial gland proliferation in the E2 treated group and the absence of growth in the placebo group (Supplemental Figure S2). Tumors grew throughout the whole uterine wall and expanded locally beyond the uterus. Histologic evaluation confirmed the orthotopic localization of EC, embedded in the endometrium/myometrium (Figure 3a). As tentative discrimination between mouse and human (i.e., induced tumours) tissues, we used immunohistochemistry and antibodies able to recognise, predominantly, the mouse ERα (MC-20) or both human and mouse ERα (HC-20; Figure 3b). While some cross reactivity between species of these antibodies is present, they indicate that the tumour is most likely of human origin. Tumor histology showed no clear glandular organization, but still it recapitulated the human situation and nested or trabecular histology structures with hypochromatic nuclei and several mitotic figures were seen (Figure 3c,d). Large tumours showed signs of necrosis (Figure 3d). No major histologic differences were seen between the used clones (not shown).

### 2.5. Metastases

**Figure 4:**
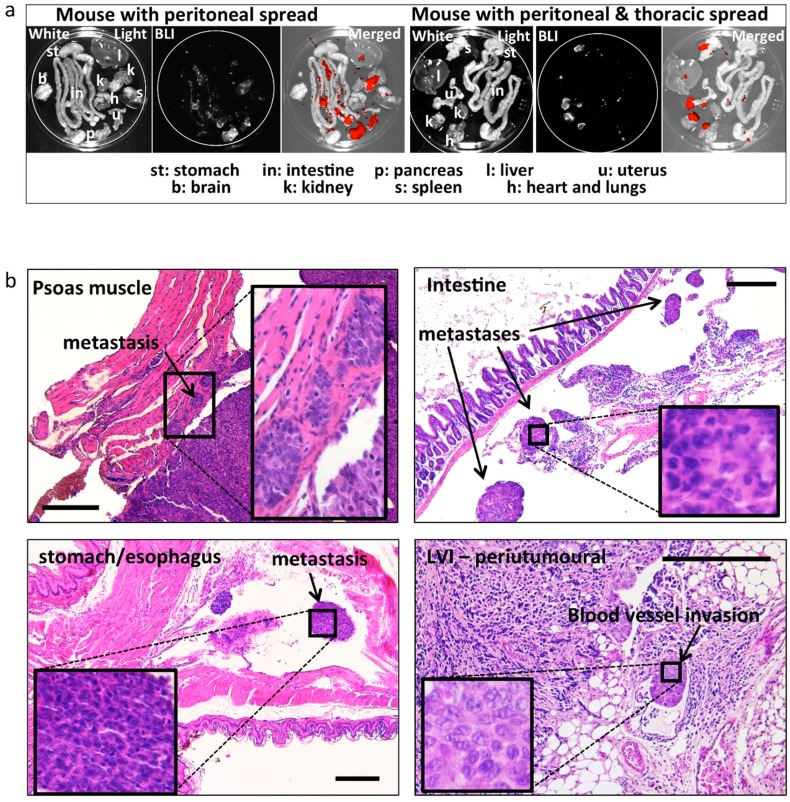
Ex-vivo BLI and histologically confirmed metastases. (**a**) Representative images of post mortem ex-vivo BLI used to assess the metastatic spread to different peritoneal and extra peritoneal organs. Left: example of spread restricted to peritoneal organs. Right: example of spread to the heart/lungs (thoracic cavity). Strong BLI signal (white in the BLI images and red in the merged images) was associated with fat tissue adjacent to peritoneal organs (spleen, kidneys). (**b**) Representative images and histologic confirmation of tumour spread to the psoas muscle, intestine, stomach/oesophagus, LVI, and infiltrating tumour cells in the (anatomic location/organs are indicated in the images). Bar scale: 300 μm.

Metastatic spread was assessed post-mortem by ex-vivo BLI, and it was observed in all mice (Table 1 and Table 2 and Figure 4a), the extent being related to the tumour size. Metastatic spread was also assessed histologically in all peritoneal organs (see Table 1 and Table 2 and Figure 4b). Slides were prepared for histology every 200 μm of thickness in order to cover the complete depth of the tissue. In most cases, metastatic peritoneal lesions were superficial on various organs in the abdomen, and with some exceptions, did not infiltrate the organs (Figure 4b). Careful histologic assessment was performed by an experienced pathologist (LK; blinded for other characteristics and origin of the samples) for the presence of lymphovascular invasion (LVI) in the tissue surrounding the uterus and in the tumour. Seven out of eight E2 treated animals (87%) developed LVI, whereas only 1/8 (12%) developed LVI in the placebo group (Table 1 and Table 2). Representative sections depicting metastases at different sites are displayed in Figure 4b.

## 3. Discussion

In this study we describe the development of an orthotopic endometrial cancer (EC) mouse model where estrogen exposure was controlled and estrogen dependent tumour growth was measured. Tumors were induced using the well-differentiated human endometrial adenocarcinoma Ishikawa cell line, modified to express a luciferase reporter gene [10] for bioluminescence imaging (BLI). Moreover, a novel MedRod delivery system was used in which subcutaneous implants provided a constant release of 17β-estradiol (E2) after ovariectomy (OVX).

Various in vitro and in vivo models of EC exist. A few EC cell lines are available and those authenticated and deposited in cell banks are the estrogen sensitive Ishikawa—the less estrogen sensitive Hec1A, Hec1b, RL95.2, KLE, and An3CA (www.atcc.org). These cells can be grown in vitro as monolayers or as spheroids [20]. It is however challenging to measure direct cell proliferation (e.g., increased cell number, BrdU incorporation) in response to E2 stimulation using these cells (own experience and [21]) and it is frequently necessary to assess cell growth using immunohistochemical markers like Ki-67 or cyclin expression. One of the simplest in vivo models is based on EC cells xenografted on the chorioallantoic membrane (CAM) of fertilised eggs [10]. This model is very suitable to test drugs with strong cytotoxic effects, thus allowing measuring differences in tumour volumes/wet-weight as endpoints [22], but due to the short time of xenograft growth (approximately five days), long-term experiments (such as the E2-dependent growth) need to rely on immunohistochemical markers as endpoints [10]. EC models based on rodents are also available in which tumours develop in mice because of genetic manipulation or chemical exposure or tumours are induced via xenotransplantation of tumour cells subcutaneously or in the fat pad [21].

A subcutaneous EC xenograft model in OVX mice receiving estrogen supplementation was also developed and showed estrogen dependency [17]. The induction of tumours orthotopically, however, represents an important advancement in oncology research, and orthotopic EC models were recently developed using Hec1a and Ishikawa cells [13,14,15]. The orthotopic model is a more biologically relevant model than the subcutaneous or fat pad models since tumours are grown in the same microenvironment and the same physiologic conditions as these occur in humans. In addition, as shown in the present study and in previous publications [13,14], the orthotopic model has clinical relevance because tumour cells infiltrate the myometrium and invade the peritoneal cavity and the vascular and lymphatic systems as well, thus mimicking the progression of human disease. In our study, we advance this orthotopic mouse model of EC to optimise and control its estrogen dependency upon OVX. Since most ECs are diagnosed and treated after menopause, when the ovaries no longer produce estrogens, correctly dosing the levels of these steroids is of extreme importance to best mimic the human EC.

In order to exogenously dose and control estrogen levels, we used a recently optimised delivery system consisting of subcutaneous implants called MedRods. This release system provides cost effective and a constant dosage of the immobilised compound by diffusion in the body for a long period (minimum 10 weeks), therefore, in most cases, only one implantation for the whole duration of the experiment is required. Different delivery systems for exogenous administration of steroids and other compounds exist, which include oral administration via drinking water, regular injections, subcutaneous pellets, or osmotic mini pumps [23,24,25]. These different delivery systems have obvious advantages and disadvantages with lab-to-lab preferences. Administration via drinking water is cost effective but dosing is difficult to control. Injections require continuity in the application, resulting in concentration peaks and causes discomfort to the animals. Subcutaneous pellets are easy to apply but often the delivery rate is not constant, whereas osmotic minipumps are efficient in terms of delivery rates, full customisation of the compound to deliver is feasible (minipumps are assembled by the investigators themselves) but are relatively expensive, large in size, and have a limited elapse time, which may require pump replacements in the course of the experiment. Using the MedRod implants, our model proved to be E2 dependent with clear differences in tumour growth and wet-weight between the E2 and placebo groups. Thus, MedRod implants provide a valid alternative to other existing delivery systems. In our experiments we used a relatively high level of E2, i.e., 300–400 pM, which is about 10 times more concentrated compared with the peak E2 levels in cycling female mice (proestrus, E2 level is about 40 pM). This E2 dose was chosen on the one hand because it was used in previous studies [16,17,19,26], and on the other hand to secure both fast tumour growth and clear and measurable differences between placebo and E2 groups. Nevertheless, lower estrogenic doses (e.g., the low active E1 at concentrations lower that 100 pM, comparable or lower than 40 pM of E2—Supplemental Figure S3, but also the experiment on cell titre performed in gonad intact mice, Table 1) elicited tumour growth and uterine responses that were indistinguishable from those induced by the positive control E2.

Since most EC in humans are estrogen dependent, it is extremely relevant that the present orthotopic EC xenograft model exhibits this feature of the human disease. The estrogen dependency of EC is currently not exploited in the clinic as is in breast or prostate cancer treatment, due to the reported low efficacy of endocrine drugs for EC patients [3,4,5,6]. While single-endocrine drug treatments have failed to show good efficacy, a recent trial of dual regimen including an aromatase inhibitor (AI) and an mTOR inhibitor yielded promising results with good efficacy [7]. It is generally agreed upon that there is room for improvement in the current use of hormonal drugs in EC, and that endocrine treatment may represent an important approach for EC in the future [27]. This is demonstrated by the numerous, currently ongoing phase II trials that are testing various combinational treatments, e.g., mTOR inhibitor in combination with AI and metformin (NCT01797523: https://clinicaltrials.gov); AI and ribociclib (NCT03008408); dual mTORC1/mTORC2 inhibitor and AI (NCT02730923); mTOR and AI inhibitors compared with progestogens and tamoxifen (NCT02228681); ribociclib and AI (NCT02657928); sodium cridanimod in conjunction with progestin (NCT03077698); and AI combined with palbociclib (NCT02730429).

In addition, a number of novel potential endocrine drug targets are being discovered [8,9,10,28,29] and therefore the establishment of proper tools for pre-clinical testing of these novel endocrine targets is of foremost importance.

The protocols and methods described in this study can help future research in endocrine drug discovery and accelerate the translation from bench to bedside. We use E2 stimulation as the reference of positive tumour growth, but the model is amenable to mimic different clinical situations, like post menopause (over 90% of all EC cases), recapitulated by OVX and ad-hoc steroid supplementation, or pre menopausal EC, using gonad intact mice with cycling E2 and progesterone. We used BLI for tumour growth monitoring, but we also demonstrated a good correlation between BLI and CE-CT findings, making our method also suitable for systems lacking an endogenous reporter gene (e.g., luciferase/BLI), such as patient derived EC tumour xenografts (PDX), the most relevant animal model in cancer research available today [30].

In conclusion, we present here an orthotopic xenograft mouse model of EC that mimics the human condition in terms of tumour localization, estrogen dependency, and metastatic spread. This model will be useful for future pre-clinical studies testing the efficacy of novel drugs and of combinational regimens of novel and existing treatments.

## 4. Materials and Methods

### 4.1. Ethics Statement

All animal procedures were approved by The Netherlands National Committee for the protection of animals used for scientific purposes and the Central Authority for Scientific Procedures on Animals or by the National Animal Experiment Board of Finland (DECNR: 2012_079, September 2012). All procedures were performed according to the European Convention for the Protection of Vertebrates Used for Scientific Purposes.

### 4.2. Cell Lines and Tumor Graft Preparation

The human endometrial adenocarcinoma cell line Ishikawa (ECACC, Sigma-Aldrich, Zwijndrecht, The Netherlands) was previously modified in our laboratory to express firefly luciferase fused with green fluorescent protein (GFP) for non-invasive bioluminescence imaging (BLI). The established Ishikawa clones were thoroughly characterized for the maintenance of markers of the parental Ishikawa cell line and were authenticated by Short Tandem Repeat (STR) profiling [10]. Since different cell lines/clones may behave differently (clonal effects), three different Ishikawa clones (Ishi-M3-HSDA, Ishi-M1-HSDB, and Ishi-M3-EVC, from now on referred to as clone 1, clone 2, and clone 3) were used in the present animal experiments. Cells were maintained in RPMI 1640 (Invitrogen, Carlsbad, CA, USA) supplemented with sodium-pyruvate, l-glutamine, penicillin-streptomycin, and 10% foetal bovine serum at 37 °C with 5% CO_2_ in humidified air, and tested negative for mycoplasma (MycoAlert, Promega, Madison, WI, USA). For orthotopic injections, cells at no more than 70% confluency were detached with Accutase (Invitrogen), pelleted and resuspended in 30 µL ice-cold Matrigel (Basement Membrane Matrix; Becton Dickinson, Vianen, The Netherlands).

### 4.3. Optimization of the MedRod Steroid Delivery System

To control estrogen exposure (for estrogen dependent tumour growth), the endogenous source of estrogens (ovaries) was removed and an exogenous estrogen supply was provided (estrogen supplementation) by using the recently optimised MedRod implants (PreclinApps Ltd., Raisio, Finland). MedRods are polydimethylsiloxane cylinders covered by a silicone membrane that have a constant release of matrix embedded 17β-estradiol (E2) for at least 10 weeks. The serum estrogen levels in MedRod bearing mice were determined by LC-MS/MS—as previously described using approximately 1 mL of blood collected at sacrifice by heart puncture [31].

### 4.4. Orthotopic Estrogen Dependent Endometrial Cancer Mouse Model

Eight-week-old female athymic nude mice (Crl:NU(NCr)-Foxn1nu) were purchased from Charles River (‘s-Hertogenbosch, The Netherlands) and housed in groups of 3–4 animals in individually ventilated cages and under specified pathogen-free conditions. Food and water were both sterilised and provided ad libitum. Complete diet with moderate energy density and very low nitrosamine content (Mouse maintenance diet, V1534-703, ssniff Spezialdiäten GmbH, Soest, Germany) was used.

For tumour cell injections, mice were anaesthetised with isoflurane (Forane, Abbott laboratories Ltd., Maidenhead, UK) using 4–5% isoflurane in pressured air for induction of sedation and 1.5% isoflurane in pressured air for maintenance. Mice were placed on a heating pad in ventral decubitus. The skin was disinfected, an incision was made laterally at the lower dorsum, and one uterine horn was exteriorised (in most cases, the left uterine horn was operated). If ovariectomy (OVX) was performed (see text), ipsilateral OVX was first performed at the uterine horn side of tumour induction. To this end, a ligature was placed in the fallopian tube and the ovary was removed above the ligature. The uterine horn was clamped at the two edges just before the fallopian tube at the top and just before the uterus body at the bottom in order to prevent leakage. Re-suspended cells (30 µL of ice-cold Matrigel; 1–5 million cells as indicated in the text) were slowly injected into the endometrial cavity with a 25 G needle. The needle was retracted after waiting for a few seconds to allow the polymerisation of the Matrigel cell-suspension and prevent leakage in the abdominal cavity. Muscle and skin incisions were sutured with 6–0 absorbable sutures (Supplemental Figure S2). The un-resected ovary was removed approximately two weeks after tumour cell injection, when the tumours had successfully engrafted (determined by BLI; next paragraph).

For the implantation of the MedRod implants containing either placebo or E2 (releasing 1.5 μg E2/day; 5.5 nmol/day), a small incision was made in the loose skin of the animal neck. A pocket was bluntly dissected under the skin and the MedRod was placed using forceps. The incision was closed with a 6–0 absorbable sutures. For analgesia, mice received 7.5 mg/kg Carprofen (Norbrook Laboratories Ltd., Newry, UK) subcutaneously, pre and post-operatively, for the following two days. The time-line of the orthotopic xenograft model is illustrated in Figure 1a.

### 4.5. Imaging by BLI

Tumor growth was visualised weekly by sequential BLI using the Andor iXon Ultra 897 camera in the X-RAD 225Cx system (Precision X-ray Inc., North Branford, CT, USA). Mice were anaesthetised with isoflurane and injected intraperitoneally with 150 mg/kg D-Luciferin (Becton Dickinson) approximately 10 min prior to imaging. Six planar images were obtained at 0, 45, 90, 180, 270, and 315° angles (angle 0° corresponding to image from the top, dorsal side of the mouse). Images obtained from angles 270°–315° were used to compute the BLI signal intensity for mice where the tumour was induced on the left uterine horn (45°–90°, in the case of right horn tumour induction). BLI data was analysed using ImageJ (v.1.48, National Institute of Health—NIH—Bethesda, MD, USA) [32], and the total photon flux was determined in the Region of Interest (ROI) located in the abdominal area corrected for background signal.

### 4.6. Micro Contrast Enhanced-Computed Tomography (CE-CT)

Prior to euthanasia, micro CE-CT imaging was performed using the small animal micro-IR (X-RAD 225Cx, Precision X-ray Inc., North Branford, CT, USA). Mice were anaesthetised with isoflurane, and to enhance soft tissue contrast, 150 µL of iodinated Omnipaque 350 (GE Healthcare, Little Chalfont, UK) was injected in the tail vein immediately prior to imaging. Images were acquired as described earlier [33]. In short, an 80 kVp, 2.5 mA imaging protocol with an acquisition rate of 5 frames/s, a spatial resolution of 100 microns, and a gantry rotation of 0.5 revolution/min was used to image the abdominal region of all animals. The imaging dose was 39 cGy, spot size was approximately 1 mm, and the imager mode was in low gain (2 × 2 binning). The beam was filtered with 2 mm aluminum to remove the low energy photons that do not contribute to the imaging.

The micro CE-CT images were analysed using the PMOD v.3.7 software (PMOD Technologies LLC, Zürich, Switzerland). The maximum tumour diameter was measured in three orthogonal planes (*x*, *y* and *z*) and the tumour volume was estimated using the following equation; tumour volume = *x* × *y* × *z*/2. Tumor density was measured in a volume of interest (VOI) placed in a representative part of the tumour, avoiding necrotic or haemorrhagic areas if present. The volume of the tumour VOIs had a median (range) of 0.02 (0.002–0.39) mL, the wide range is explained by the variable tumour size.

### 4.7. Histological Examination and Immunohistochemistry

Tissue biopsies were fixed in 3.7% formaldehyde, embedded in paraffin and processed for histological examination. Histology was determined by a pathologist (LK) from 4 µm haematoxylin & eosin (Sigma-Aldrich, Zwijndrecht, The Netherlands) stained sections. Detection of estrogen receptor-α (ERα) of human origin was performed with immunohistochemistry using the antibody HC-20 (Santa Cruz Biotechnologies, Heidelberg, Germany), whereas for mouse ERα, antibody MC-20 (Santa Cruz Biotechnologies) was used. In brief, sections were subjected to deparaffinization followed by rehydration. Heat-induced antigen retrieval was performed and slides were blocked in 1% BSA/PBST and subsequently incubated overnight at 4 °C with 500 times diluted ERα antibody, as described earlier [10]. The EnVision detection system was used according to the manufacturer’s manual followed by visualization with Diaminobenzidine (DAB).

### 4.8. Statistics

Statistical analyses were performed using KaleidaGraph version 4.1.3. (Synergy Software, http://www.synergy.com). Variance was analysed using parametric student’s *t*-test whereas the Pearson’s correlation coefficient was used to evaluate the relationship between two variables. Differences and correlations were considered statistically significant at *p* < 0.05.

## Figures and Tables

**Table 1:** Optimization of cell titre for tumour induction.

Injected Cells (Number)	Number of Mice	Mice with Tumor (%)	Peritoneal Metastases *	Distant Metastases ^#^
1 × 10^6^	3	2 (67%)	I	0
3 × 10^6^	3	3 (100%)	I, L, K, S, St	1 (T + LVI)
5 × 10^6^	3	3 (100%)	I, L, K, S, St	0

* All mice developed peritoneal spread as assessed by ex-vivo BLI (bioluminescence). ^#^ Number of mice with metastases. I = intestine, L = liver, K = kidney, S = spleen, St = Stomach, T = thoracic cavity; LVI = lymphovascular invasion.

**Table 2:** Overview of the experimental animals used to develop the E2-dependent endometrial cancer model.

Ishikawa (3 × 10^3^ Cells)	OVX	Placebo/E2	Number of Mice	Mice with Tumor	CE-CT	Peritoneal Metastases *	LVI ^#^ (No.)
Clone 1	+	Placebo	3	3 (100%)	3	I, L, K, S (P, A)	0
	+	E2	3	3 (100%)	2	I, L, K, S, St (P, A)	3
Clone 2	+	Placebo	2	2 (100%)	2	I, L, K, St (A)	1
	+	E2	3	3 (100%)	3	I, L, K, S, St (P, A)	2
Clone 3	+	Placebo	3	3 (100%)	0	(A, P)	0
	+	E2	2	2 (100%)	0	(A)	2

* All mice developed peritoneal spread as assessed by ex vivo BLI. Location is indicated as follow: I = intestine, L = liver, K = kidney, S = spleen, St = stomach, P = psoas, A= adipose tissue (by brackets, histologically confirmed); ^#^ LVI = lymphovascular invasion; number of mice with metastases.

**Table 3:** Overview of the CE-CT scan data.

Ishikawa	Placebo/E2	*x* (cm) ^1^	*y* (cm) ^2^	*z* (cm) ^3^	Tumor Volume (mL) ^4^	Tumor Density ± SD ^5^	Tumor Weight *
Clone 1	placebo	n.d.	n.d.	n.d.	n.d.	n.d.	n.d.
Clone 1	placebo	0.27	0.23	0.27	0.01	131.7 ± 15.6	20
Clone 1	placebo	0.63	0.53	0.41	0.07	−9.3 ± 4.9	118
Clone 2	placebo	0.14	0.14	0.23	0.002	137.3 ± 25.9	18
Clone 2	placebo	1.18	0.9	1.47	0.78	107.8 ± 4.5	568
Clone 2	E2	0.68	0.74	0.93	0.23	24.8 ± 4.7	333
Clone 1	E2	0.57 + 0.80 + 1.0	0.51 + 0.94 + 0.76	0.63 + 0.95 + 0.85	0.77	57.3 ± 8.0	1123
Clone 1	E2	0.78	0.98	0.97	0.37	31.2 ± 3.2	353
Clone 2	E2	n.d.	n.d.	n.d.	n.d.	n.d.	2147
Clone 2	E2	1.25	1.04	1.58	1.03	47.8 ± 15.2	1347
Clone 2	E2	0.49	0.45	0.43	0.05	−6.3 ± 10.4	81 ^6^

^1^*x*: Maximum transverse tumour diameter at the axial image depicting the largest tumour diameter. ^2^*y*: Maximum anterioposterior tumour diameter at the axial image depicting the largest tumour diameter. ^3^*z*: Maximum sagittal tumour diameter at the sagittal image depicting the longest sagittal tumour diameter. ^4^ Tumor volume (mL): = *x* × *y* × *z*/2. * Tumor weight: real wet-weight (in mg) of surgically removed tumours after sacrifice. n.d.: non-detectable/determinable. ^5^ Tumor density is indicated as mean values ± standard deviation (SD). ^6^ This mouse (indicated by the red dot in Figure 2) belongs to the E2 treated group but initially there was no tumour engraftment (i.e., no BLI signal at the moment of E2 supplementation, week 0, probably due to sub-optimal cell injection). This mouse was nevertheless kept in the experiment and showed BLI signals, though strongly delayed, during the next measurements.

## Supplementary Materials

Supplementary materials can be found at http://www.mdpi.com/1422-0067/19/9/2547/s1.

## Abbreviations

BLI	Bioluminescence imaging
CAM	Chorioallantoic membrane assay
CE-CT	Contrast-enhanced computerised axial tomography
E2	17β-Estradiol
EC	Endometrial cancer
GFP	Green fluorescent protein
LC-MS/MS	Liquid chromatography tandem mass spectrometry
LVI	Lymphovascular invasion
OVX	Ovariectomy
ROI	Region of Interest
