# Supplementary material for: Development of an Image-Guided Orthotopic Xenograft Mouse Model of Endometrial Cancer with Controllable Estrogen Exposure
Source: Int J Mol Sci. 2018 Aug 28;19(9):2547. doi: 10.3390/ijms19092547 (PMC6165149; doi:10.3390/ijms19092547)
Supplement: Supplementary file 1 [file ijms-19-02547-s001.pdf]

International Journal of
Molecular Sciences

MDPI

Supplementary

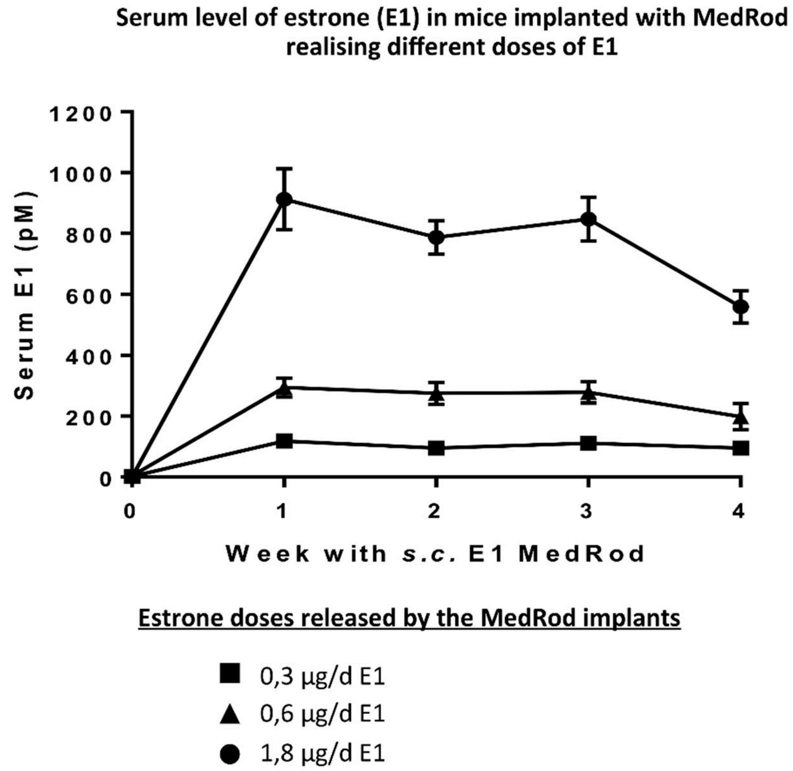

Figure S1. Optimisation of the MedRod delivery using estrone as test compound. Serum levels of the estrogenic compound estrone (E1) measured by LC-MS/MS in athymic female mice implanted subcutaneously (s.c.) with MedRod releasing E1 at different doses, i.e., 0.3 µg/d (squares), 0.6 µg/d (triangles) or 1.8 µg/d (circles). Each line represents data from 5 mice. Data is expressed as mean ± SEM.

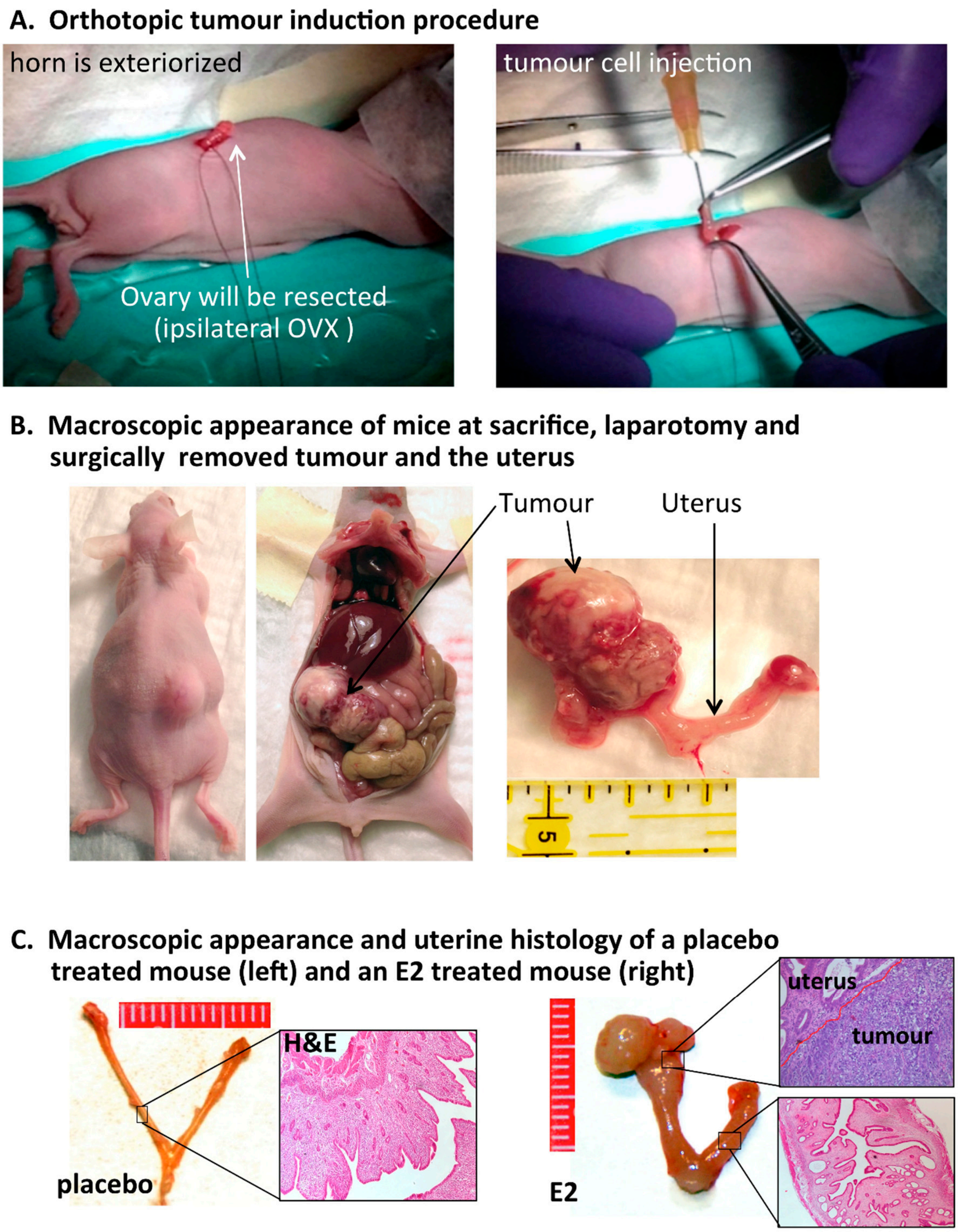

Figure S2. Overview of the tumour induction and characterisation. (**a**). Procedure of orthotopic tumour induction. One uterine horn is exteriorized and ipsilateral OVX was performed prior to cell injection. Cells resuspended in 30 µl ice-cold Matrigel are then slowly injected through the myometrium, into the endometrial cavity with a 25G needle syringe. (**b**). Macroscopic appearance of mice, abdominal cavity at laparotomy and surgically resected uterus with tumour at the time of sacrifice. (**c**). Macroscopic appearance of uterus with tumour and histology (haematoxylin & eosin, H&E) of mouse endometrium receiving either placebo (no growth/atrophic endometrium) or E2 (with evident glandular growth).

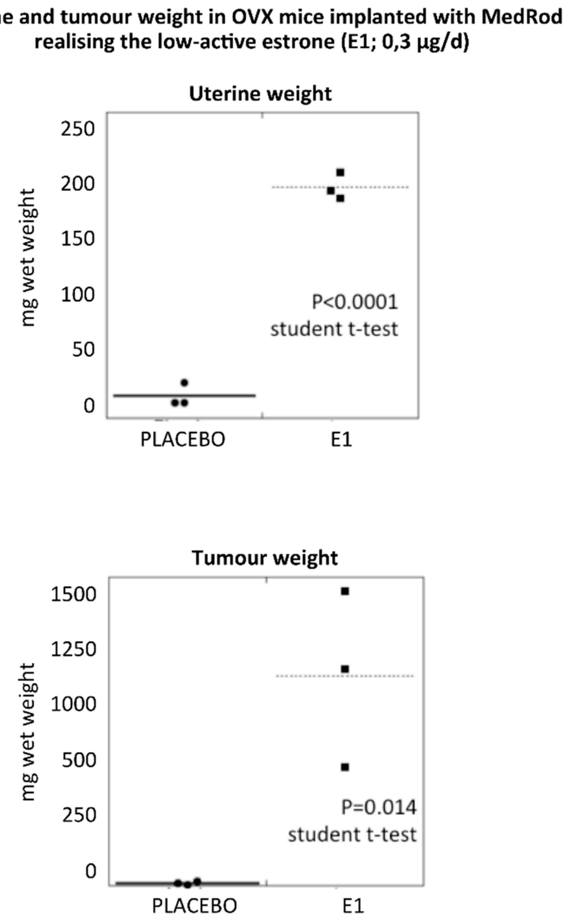

Figure S3. Uterine and tumour weight using less-sustained estrogen signalling.

Since experiments were conducted with high E2 doses, uterine and tumour growth were tested using pellets (MedRods) releasing the low active E1 at a dose as low as of 0.3 µg/day corresponding to approximately 80nM of circulating E1 (see supplemental Figure 1). E1 requires concentrations of at least 1-2 magnitudes higher than E2 to elicit similar responses [33]. Data shown in figure refers to the wet weights at humane endpoint (animal sacrifice, six weeks after OVX). E1 was not significantly converted to E2 (measured by LC-MS in these mice) but it induced strong uterine responses and tumour growth that were not distinguishable from E2 treated mice.
